# Efficacy of premedication with intranasal dexmedetomidine for removal of inhaled foreign bodies in children by flexible fiberoptic bronchoscopy: a randomized, double-blind, placebo-controlled clinical trial

**DOI:** 10.1186/s12871-019-0892-6

**Published:** 2019-12-02

**Authors:** Yanmei Bi, Yushan Ma, Juan Ni, Lan Wu

**Affiliations:** 1 Department of Anesthesiology, West China Second University Hospital, Sichuan University, Chengdu, Sichuan Province China; 2 Key Laboratory of Birth Defects and Related Diseases of Women and Children (Sichuan University), Ministry of Education, Chengdu, Sichuan Province China

**Keywords:** Foreign body, Fiberoptic bronchoscopy, Dexmedetomidine

## Abstract

**Background:**

Tracheobronchial foreign body aspiration in children is a life-threatening, emergent situation. Currently, the use of fiberoptic bronchoscopy for removing foreign bodies is attracting increasing attention. Oxygen desaturation, body movement, laryngospasm, bronchospasm, and breath-holding are common adverse events during foreign body removal. Dexmedetomidine, as a highly selective α_2_-adrenergic agonist, produces sedative and analgesic effects, and does not induce respiratory depression. We hypothesized that intranasal dexmedetomidine at 1 μg kg − 1 administered 25 min before anesthesia induction can reduce the incidence of adverse events during fiberoptic bronchoscopy under inhalation general anesthesia with sevoflurane.

**Methods:**

In all, 40 preschool-aged children (6–48 months) with an American Society of Anesthesiologists physical status of I or II were randomly allocated to receive either intranasal dexmedetomidine at 1 μg·kg − 1 or normal saline at 0.01 ml kg^− 1^ 25 min before anesthesia induction. The primary outcome was the incidence of perioperative adverse events. Heart rate, respiratory rate, parent-child separation score, tolerance of the anesthetic mask, agitation score, consumption of sevoflurane, and recovery time were also recorded.

**Results:**

Following pre-anesthesia treatment with either intranasal dexmedetomidine or saline, the incidences of laryngospasm (15% vs. 50%), breath-holding (10% vs. 40%), and coughing (5% vs. 30%) were significantly lower in patients given dexmedetomidine than those given saline. Patients who received intranasal dexmedetomidine had a lower parent–child separation score (*P* = 0.017), more satisfactory tolerance of the anesthetic mask (*P* = 0.027), and less consumption of sevoflurane (38.18 ± 14.95 vs. 48.03 ± 14.45 ml, *P* = 0.041). The frequency of postoperative agitation was significantly lower in patients given intranasal dexmedetomidine (*P* = 0.004), and the recovery time was similar in the two groups.

**Conclusions:**

Intranasal dexmedetomidine 1 μg·kg^− 1^, with its sedative and analgesic effects, reduced the incidences of laryngospasm, breath-holding, and coughing during fiberoptic bronchoscopy for FB removal. Moreover, it reduced postoperative agitation without a prolonged recovery time.

**Trail registration:**

The study was registered with the Chinese Clinical Trial Registry (registration number: ChiCTR1800017273) on July 20, 2018.

## Background 

Tracheobronchial foreign body (FB) aspiration in children may be a life-threatening, emergent situation [1]. Undiagnosed or delayed treatment of a tracheobronchial FB may result in pneumonia, atelectasis, a lung abscess, or fatal airway obstruction [2–4]. Prompt, successful removal of an FB is associated with fewer complications and deaths [5, 6]. Rigid bronchoscopy is the main diagnostic and therapeutic procedure for patients suspected to have aspirated a foreign body. It allows an excellent control of the airway, provides a large working channel and permits the removal of foreign bodies and thick mucus plug. The use of fiberoptic bronchoscopy to remove tracheobronchial FBs is currently attracting increased attention [7, 8]. The flexible bronchoscopy compared with the rigid bronchoscope is relatively atraumatic, allows the visualization of the upper lobes as well as the natural dynamics of the palate and larynx. The procedure is performed via a laryngeal mask airway (LMA) under general anesthesia. Oxygen desaturation, body movements, laryngospasm, bronchospasm, and breath-holding are common adverse events during FB removal [2, 9].

Dexmedetomidine, a highly selective α_2_-adrenergic agonist, provides sedation without respiratory depression. Used as a preoperative medication, it reduces preoperative anxiety [10, 11], lowers the anesthetic requirement, and deepens the level of anesthesia [12, 13]. Several studies have evaluated the sedative effect of intravenous infusion of dexmedetomidine during fiberoptic bronchoscopy and confirmed that this agent is useful for reducing intratracheal stimuli (by decreasing the incidence of coughing, breath-holding, and laryngospasm) and enhancing patients’ degree of comfort without the risk of respiratory depression [14–16]. Nevertheless, the patient’s recovery time is significantly prolonged by intravenous infusion of dexmedetomidine [15]. It has been reported that the plasma concentrations of dexmedetomidine approaches 100 pg·ml^− 1^ (the low end reported for sedative efficacy) within 20 min of intranasal administration of atomized dexmedetomidine 1 μg·kg^− 1^ in children [17], thereby producing satisfactory sedation before anesthesia induction [18]. The effect of premedication with intranasal dexmedetomidine on reducing the incidence of adverse events during flexible bronchoscopy in children, however, remains undetermined.

This prospective, randomized, double-blind, placebo-controlled study was performed to evaluate whether intranasal dexmedetomidine at a dose of 1 μg·kg^− 1^ administered 25 min before anesthesia induction can reduce the incidence of adverse events during fiberoptic bronchoscopy under sevoflurane inhalation general anesthesia.

## Methods

This study adheres the applicable CONSORT guidelines. This prospective, randomized, double-blind, placebo-controlled, single-center clinical trial was conducted at the West China Second University Hospital (Sichuan University, Chengdu, Sichuan Province, China). The study was registered with the Chinese Clinical Trial Registry (#ChiCTR1800017273). The China Ethics Committee of Registering Clinical Trials approved the study protocol (#ChiECRCT-20180113). The parents or legal guardians of each patient were supplied with comprehensive information by one of the investigators, regarding the study’s risk, objectives, and procedures. The parents/legal guardians signed informed consent before the patient’s inclusion in the study.

### Patients

We enrolled 40 children (age 6–48 months) whose American Society of Anesthesiologists physical status was I or II and who were undergoing FB removal via fiberoptic bronchoscopy during the period from August 10 to December 25, 2018. Patients with congenital disease, a family history of malignant hyperthermia, coagulation disorders, asthma, severe preoperative respiratory impairment (i.e., single-lung emphysema or other type of severe atelectasis), and/or allergy to anesthetics were excluded from the study.

**Fig. 1:**
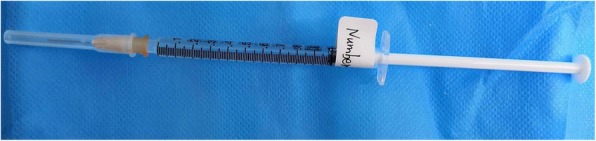
Dexmedetomidine 100 μg·ml^−1^ or 1-ml normal saline in 1-ml syringe ready for intranasal administration

In preparation, all patients fasted from solids for 6 h, breast milk for 4 h, and clear fluids for 2 h before intervention. They were premedicated with atropine at 10 μg·kg − 1 i.v. 30 min before the induction of anesthesia. The patients were randomly assigned to one of two groups (Dexmedetomidine (DEX) group and control group) using a simple computerized concealed-envelope method. At 25 min before anesthesia induction, the patients were administered either intranasal dexmedetomidine (20,171,202; Nhwa Pharmaceutical Co., Ltd., Jiangsu, China) 1 μg·kg^− 1^ (100 μg in 1 ml) or intranasal normal saline 0.01 ml·kg^− 1^ (Fig. 1). The intranasal drugs were prepared by a dispensing nurse of our department, then administered by a doctor who was unware of patient randomization.

### Fiberoptic bronchoscopy

**Fig. 2:**
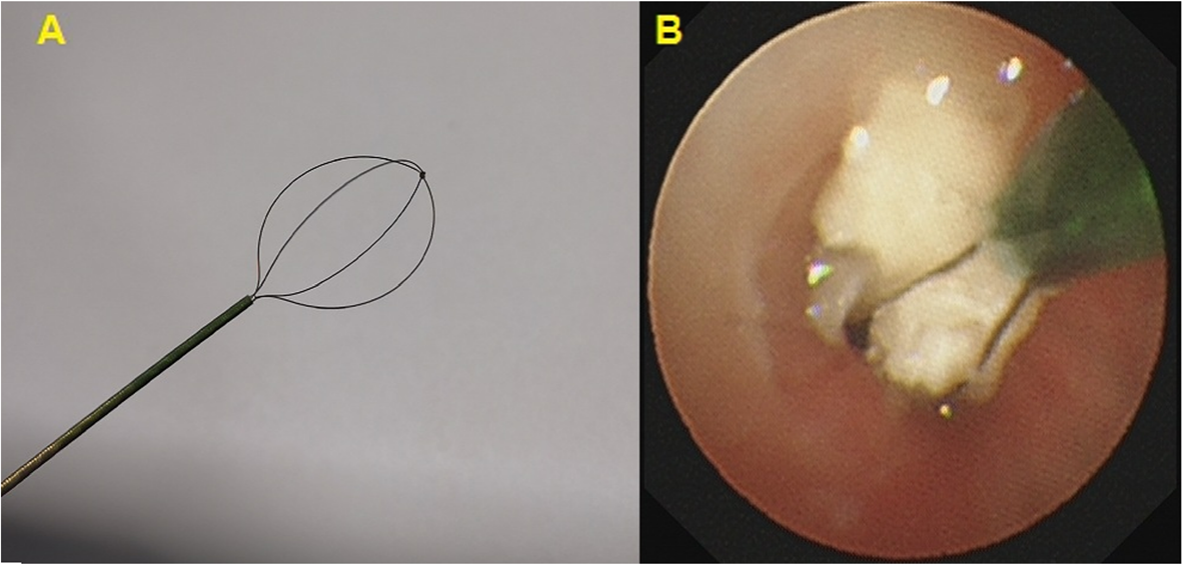
Foreign body basket used for foreign body removal. **a** Foreign body basket. **b** Foreign body was caught in a foreign body basket

Anesthesia was induced via mask using 5–8% sevoflurane in 100% oxygen at 6 L·min^− 1^ until the BIS decreased to 40 or 4 mins after consciousness extinction and EtSevo concentration maintained at the same level (≥1.3 MAC), at which point the LMA (Henan Tuoren Medical Equipment CO., Ltd.; common LMA-classic) was inserted. Anesthesia was maintained using 3–6% sevoflurane in fresh gas at 4 L·min^− 1^ with the BIS at 40–60. The external diameters of the two widely used flexible bronchoscopes for FBs removal were 2.8 mm and 4.0 mm, respectively. At the beginning of the procedure, lidocaine 2 mg·Kg^− 1^ was sprayed on the epiglottis and larynx. FBs were removed in an FB basket (Boston Scientific Corporation; Zero Tip™ Airway Retrieval Basket; OD 1.0 mm) through the bronchoscope’s suction channel, the sizes of the channels were 1.2 mm and 2.0 mm for 2.8 mm and 4.0 mm bronchoscopies (Fig. 2). At the end of the procedure, before withdrawing the fiberoptic bronchoscope from the trachea, acetylcysteine was sprayed into the trachea via the bronchoscope. Sevoflurane was discontinued after completion of the procedure, and the patient was allowed to spontaneously breathe 100% oxygen at 6 L·min^− 1^. The LMA was removed when the patient moved spontaneously or exhibited a jaw thrust. After removing the LMA, the child was transferred to the postoperative care unit (PACU) for recovery, where he or she was given oxygen at 4–6 L·min^− 1^ via mask, and underwent heart rat (HR) and oxygen saturation (SpO_2_) monitoring. The patient was discharged from the PACU when the SpO_2_ had stabilized at > 92% for 10 min on room air.

### Monitoring

Routine patient monitoring included various measurements, including SpO_2_, respiratory rate (RR), HR, end-tidal carbon dioxide (EtCO_2_), and end-tidal sevoflurane (EtSevo). Additionally, each patient was monitored for his/her BIS (A-2000; Aspect Medical Systems, Norwood, MA, USA). The EtCO_2_ was measured by a capnography sensor placed between the L-piece and Bain circuit. The Etsevo was measured by side-stream sensor placed at the breathing circuit filter. The Gas Man anesthesia simulator (Med Man Simulations, Boston, MA, USA) was used to calculate the sevoflurane consumption.

Before induction, the HR, RR, and SpO_2_ were recorded at baseline (time 0, or T_0_). The HR, RR, SpO_2_, and BIS were then recorded at the following time points: LMA insertion (T_LMAi_), fiberoptic bronchoscope insertion (T_bron_), 5 min after beginning the procedure (T_5min_), the end of the procedure (T_end_), at LMA removal (T_LMAR_), 5 min after LMA removal (T_LMAR5_), and at discharge from the PACU (T_dis_).

### Outcome measurements

The primary outcome measurements were the incidence of adverse events including: oxygen desaturation, CO_2_ retention, coughing, body movements, bronchospasm, laryngospasm, breath-holding during the procedure, and coughing in the PACU. Oxygen desaturation was defined as SpO_2_ < 90% for 10s. CO_2_ retention was defined as EtCO_2_ ≥ 45 mmHg at the end of the procedure. Emergency treatment measures are shown in Table 1.

**Table 1:** Emergency treatment for adverse events

Adverse events	Emergency treatment
Laryngospasm	Immediately remove the fiberoptic bronchoscope
	Continuous positive airway pressure at 10cmH_2_O
	2 mg·kg^− 1^ Propofol iv.
	1 mg·kg^− 1^ Suxamethonium iv.
Bronchospasm	10μg Adrenaline iv.
Body movement	2 mg·kg^−1^ Propofol and 1 μg·kg^− 1^ remifentanil iv.
Coughing	2 mg·kg^− 1^ Propofol and 1 μg·kg^− 1^ remifentanil iv.
Breath-holding	Manual positive-pressure ventilation
Oxygen desaturation	Increase inhaled oxygen concentration
	Manual positive-pressure ventilation
Carbon dioxide retention	Mechanical ventilation

The secondary outcome measurements were (1) the separation score at the time of separating the patient from their parents and entrance into the operation room, tolerance of the anesthetic mask during anesthesia induction, the agitation score of each patient in the PACU (Table 2) [19]; (2) consumptions of sevoflurane and other extra medications; (3) anesthesia induction time, Extubation time, and recovery time. *Anesthesia induction time* was defined as the time from beginning induction to LMA insertion. *Extubation time* was defined as the time from discontinuing the sevoflurane to LMA removal. *Recovery time* was defined as the time from discontinuing of sevoflurane to opening of the eyes either spontaneously or by vocal command. All outcome parameters were recorded by another doctor who was unaware of patient randomization.

**Table 2:** Clinical scales used for the study

Separation score [19]	
1. Excellent; separate easily	
2. Good; not clinging, whimpers, easy to calm	
3. Fair; not clinging, cries, not calm with reassurance	
4. Poor; crying, clinging to their parent	
Tolerance of the anesthetic mask during anesthesia induction [19]	
1. Excellent; unafraid, cooperative, easy acceptance of mask	
2. Good; slight fear of mask, easy to quite	
3. Fair; moderate fear, not quite with reassurance	
4. Poor; terrified, crying, agitated	
Agitation score [19]	
1. Sleeping	
2. Awake, calm, and cooperative	
3. Crying, need consolation	
4. Restless, screaming inconsolable	
5. Combative, disoriented, trashing	

An agitation score of 4–5 is considered as agitation

### Sample size calculation

The sample size was calculated based on the ability to detect a 44.4% reduction in the incidence of laryngospasm with dexmedetomidine premedication (55.6% vs 11.1%, according to our preliminary study) with 80% power. The level of significance was set at two-sided α = 0.05. It was then concluded that the sample size required to achieve a statistically significance was 20 samples for each group.

### Statistical analysis

A t-test and Wilcoxon’s rank-sum test were used to access continuous variables, and the 휒^2^ test to assess categorical variables. The statistical analysis was performed with SPSS software, version 20.0 (IBM Corp., Armonk, NY, USA), *P* < 0.05 was considered to indicate statistical significance.

## Results

**Fig. 3:**
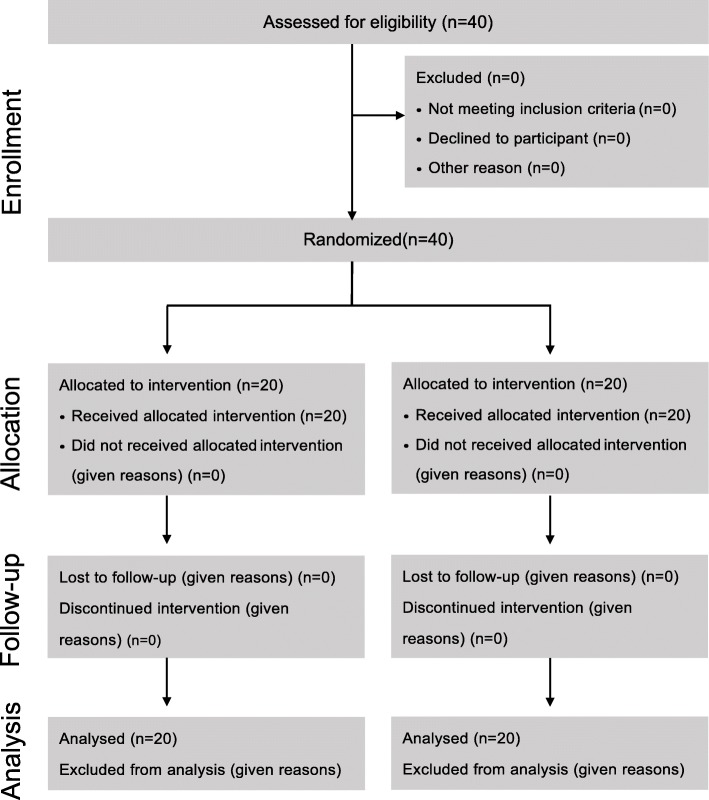
CONSORT flow diagram

Altogether, 40 patients were screened, underwent randomization, and completed the study protocol (Fig. 3). There were no differences in patients’ characteristics between the two groups except that the HR was significantly lower in patients who were given intranasal dexmedetomidine rather than saline (136 ± 21 vs. 151 ± 14 beats per minute, respectively; *P* = 0.015) (Table 3). All of the FBs were organic (walnuts, peanuts, sunflower seeds, melon seeds, raisins, and pears).

**Table 3:** Demographic characteristics

Variables	DEX Group	Control Group	*P* value
Age (months)	17.2 ± 6.3	18.0 ± 6.6	0.737
Sex (male/female)	15/5	14/6	0.723
Weight (Kg)	10.9 ± 2.2	10.8 ± 1.2	0.965
Site of foreign body (T/RB/LB/BB)	1/11/6/2	0/10/9/1	0.576
Duration of foreign body aspiration (days)	5.5(2.3–10.0)	6.0(3.3–10.8)	0.583
Time-lag between diagnosis and retrieval of foreign body (days)	2(1–3)	2(1–3)	0.904
Complications			
Obstructive emphysema	11 (55)	16(80)	0.091
Pneumonia	18 (90)	20 (100)	0.147
Atelectasis	4 (20)	1 (5)	0.151
Baseline value			
Heart rate (beats per minute)	136 ± 21	151 ± 14	0.015
Respiratory rate (beats per minute)	37 ± 9	37 ± 5	0.911
Oxygen saturation (%)	100(100–100)	100(100–100)	0.583

Date are expressed as mean ± standard deviation, median (interquartile range) number of patients (percentage). T, tracheal; RB, right bronchus; LB, left bronchus; BB, both right and left bronchus. Duration of foreign body aspiration: time from foreign body aspiration to its removal

**Fig. 4:**
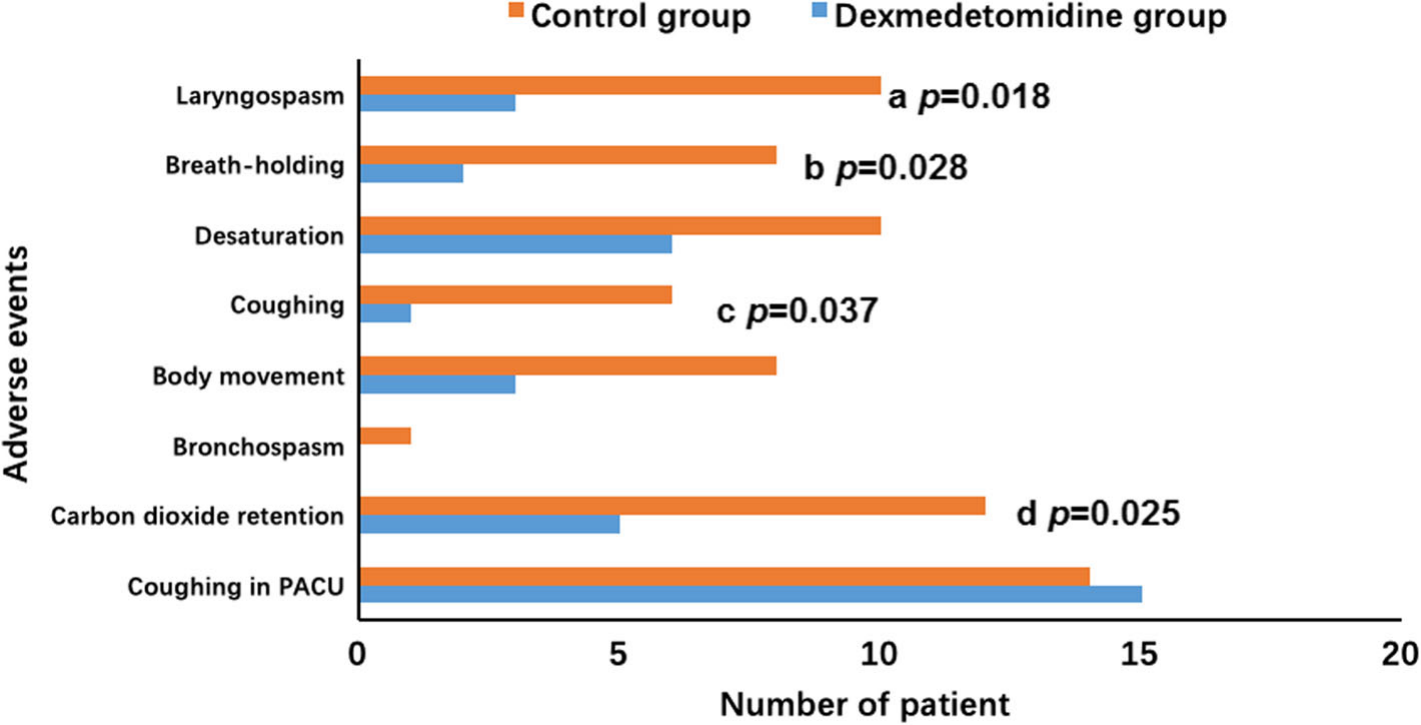
Incidence of adverse events

Compared with those given saline, the patients given dexmedetomidine had significantly lower incidences [odds ratio (95% confidence interval)] of laryngospasm [15% vs. 50%; 0.176 (0.039–0.797); *P* = 0.018], breath-holding [10% vs. 40%; 0.176 (0.030–0.924), *P* = 0.028], and coughing [5% vs. 30%; 0.123 (0.013–1.138); *P* = 0.037] (Fig. 4). The incidence of oxygen desaturation and coughing in the PACU was similar in the two groups.

**Fig. 5:**
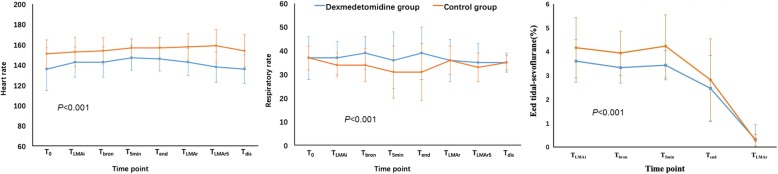
HR, RR, and Etsevo level at various time points during the study period. T_0_, baseline level before anesthesia; T_LMAi_, LMA insertion; T_bron_, begin of fiberoptic bronchoscopy; T_5min_, 5 min after beginning the procedure; T_end_, the end of the procedure; T_LMAr_, LMA removal; T_LMAR5_, 5 min after LMA removal

The RR remained more stable in patients given dexmedetomidine (*P* < 0.001) (Fig. 5). In contrast, the RR was lower in the control group during the procedure, and the controls recovered postoperatively. The incidence of CO_2_ retention was significantly lower in DEX group than in the control group (25% vs. 60%, respectively; OR = 0.222, 95% CI = 0.058–0.858; *P* = 0.025). The mean HR was lower in the DEX group (*P* < 0.001).

**Fig. 6:**
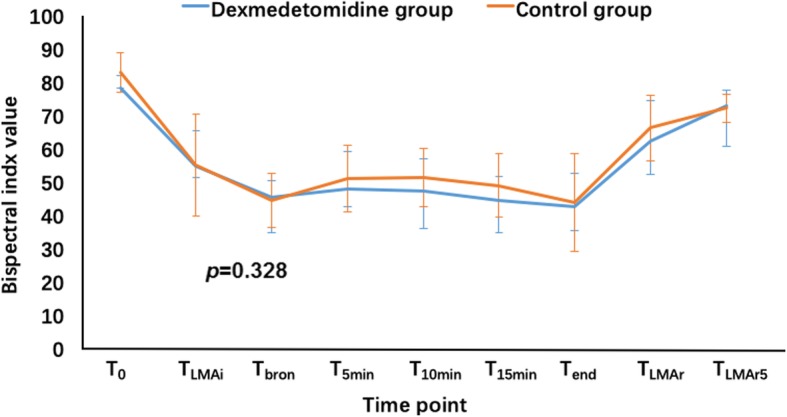
Bispectral index at various time points during the study period. T_0_, baseline level before anesthesia; T_LMAi_, LMA insertion; T_bron_, begin of fiberoptic bronchoscopy; T_5min_, 5 min after beginning the procedure; T_10min_, 10 min after beginning the procedure; T_15min_, 15 min after beginning the procedure; T_end_, the end of the procedure; T_LMAr_, LMA removal; T_LMAR_

The preoperative separation scores were significantly lower in the DEX group than the control group (*P* = 0.017) (Table 4). Patients receiving dexmedetomidine had better tolerance of the anesthetic mask (*P* = 0.027) and required less time for anesthesia induction (*P* = 0.015). The BIS values of the patients during the procedure were similar in the two groups (*P* = 0.328) (Fig. 6). EtSevo was significantly lower in the DEX group than the control group (*P* < 0.001) (Fig. 5). Consumption of sevoflurane, the agent that maintained anesthesia, was significantly lower in patients receiving dexmedetomidine (38.18 ± 14.95 vs. 48.03 ± 14.45 ml, respectively; *P* = 0.041). The number of patients need for rescue agents such as propofol and remifentanil was reduced by premedication with intranasal dexmedetomidine (*P* = 0.003 and *P* = 0.008, respectively) (Table 5).

**Table 4:** Clinical scales

Variables	DEX Group	Control Group	*P* value
Separation score2/3/4	9/9/2	2/10/8	0.017
Tolerance of anesthetic mask 2/3/4	2/9/9	1/2/17	0.027
Agitation score 2/3/4/5	8/7/4/1	1/5/10/4	0.017

Data are expressed as number of patients

**Table 5:** The characteristics and outcome of the fiberoptic bronchoscopies

Variables	DEX Group	Control Group	*P* value
Size of LMA	2(2–2)	2(2–2)	0.574
Size of fiberoptic scope (mm)	4.0(4.0–4.0)	4(4.0–4.0)	0.637
Duration of anesthesia induction (min)	6(5–6.8)	7(5–10)	0.015
Duration of procedure (min)	13.5(10.3–20)	17(11–23.8)	0.738
Extubation time (min)	6.5(4.3–9.8)	6(4–9)	0.758
Recovery time (min)	16(8.3–28.8)	11(9–22)	0.445
Propofol			
No. (%)	3(15%)	12(60%)	0.003
Dosage (mg)	30(15–35)	20(20–27.5)	0.734
Succinylcholine			
No. (%)	3(15)	4(20%)	0.677
Dosage (mg)	10(10–10)	10(10–25)	0.629
Remifentanil			
No. (%)	1(5%)	8(40%)	0.008
Dosage (ug)	10(10–10)	15(10–23.75)	0.667

Data are expressed as median (interquartile range), number of patients (percentage)

The extubation time and recovery time were similar in the two groups (*P* = 0.758 and *P* = 0.445, respectively). Agitation during recovery occurred in 25% (*n* = 5) of patients in the DEX group and 70% (*n* = 14) in the control group (*P* = 0.004). The agitation scores were significantly lower in patients premediated with dexmedetomidine (*P* = 0.017).

## Discussion

Our principal finding was that intranasal dexmedetomidine at a dose of 1 μg·kg^− 1^ given 25 min before anesthesia induction could reduce the incidence of laryngospasm, breath-holding, and coughing during fiberoptic bronchoscopy for FB removal in children. Furthermore, intranasal dexmedetomidine was associated with lower parent–child separation scores, frequency of agitation, and agitation scores. Moreover, it did not prolong the recovery time.

Dexmedetomidine uniquely provides sedative and analgesic effects without respiratory depression [14, 20, 21], even when administered at doses higher than recommended for sedation [22]. These properties render dexmedetomidine a potentially useful drug during airway surgery. Dexmedetomidine infusion given to remove an airway FB removal attenuates the airway response to fiberoptic bronchoscopy similar to remifentanil [15]. Dexmedetomidine also attenuates the airway response to endotracheal extubation [23, 24].

We also observed a lower incidence of laryngospasm, breath-holding, and coughing during fiberoptic bronchoscopy in patients given dexmedetomidine, suggesting that intranasal dexmedetomidine relieves intratracheal and laryngeal stimuli during this procedure. This effect is possibly mediated via its sedative and analgesic properties. Dexmedetomidine provides analgesia via receptors in the spinal cord, and attenuation of the stress response [25]. As shown in previous studies [10, 11], we also found that premedication with intranasal dexmedetomidine reduced the patients’ separation anxiety and resulted in more satisfactory tolerance of the facial mask during anesthesia induction. Reduction in the secretions as a result of less crying during patient separation from the parents and also during induction of anesthesia can reduce the incidence of laryngospasm and coughing.

In contrast to previous reports [23, 24, 26], we observed a similar incidence of oxygen desaturation and coughing in the PACU in our two study groups. The time from FB aspiration to its removal was similar in the two groups. This similar time lag might cause a similar incidence of pre-procedure pneumonia. Preoperative pneumonia increases respiratory tract secretions, which causes intraoperative hypoxemia, and an increased incidence of coughing in the PACU. The similar incidences in postoperative coughing may have been associated with the intra-tracheal use of acetylcysteine during the procedure.

Similar to a previous study [15], the RR was more stable in patients given dexmedetomidine. In addition, the lower incidence of CO_2_ retention indicated that dexmedetomidine did not impair the respiratory drive. We observed a lower RR in the control group during the procedure, which must have been associated with inhalation of a higher concentration of sevoflurane and/or greater consumption of propofol and remifentanil. The Et-Sevo was significantly higher in the control group during the procedure, RR decreased as the concentration of sevoflurane increased [27]. Propofol inhibits respiration by acting on GABA receptors [28, 29], whereas remifentanil produces analgesia and respiratory depression by acting on μ receptors. Moreover, the respiratory rate, CO_2_ retention, and oxygen saturation are generally maintained during dexmedetomidine sedation in children [30–32].

Compare with the control group, the lower HR during the study period in the DEX group might be explained by the decreased sympathetic outflow and circulating levels of catecholamines caused by dexmedetomidine [33].

In the present study, intranasal dexmedetomidine did not significantly prolong the patients’ recovery time, but it did significantly reduce the incidence of postoperative agitation. Emergence agitation occurs frequently in children during recovery from sevoflurane anesthesia. Postoperative restlessness is associated with a risk of self-injury and is a source of stress for both caregivers and family members. Dexmedetomidine has been used in the management of postoperative agitation because of its sedative and analgesic effects [34].

This new anesthetic agent, dexmedetomidine used alone at clinical doses, has not induced neurotoxicity in juvenile animal models [35, 36]. It exhibits neuroprotective effects in vitro and attenuates neuro-apoptosis caused by other anesthetic agents, [37, 38]. It is thus considered one of the rare “neuro-safe” anesthetic agents [39] used in infants.

There were few limitations in our study. Firstly, we used only a single dose of dexmedetomidine and thus did not compare the effects of different doses. Yuen et al., however, in a study of patients < 4 years of age, showed that intranasal dexmedetomidine 1 μg·kg^− 1^ had sedative effects similar to 2 μg·kg^− 1^ [21]. Indeed, our preliminary results showed that dexmedetomidine 1 μg·kg^− 1^ produces a satisfactory sedative effects without prolonged recovery time, whereas a 2 μg·kg^− 1^ or higher dose of dexmedetomidine significantly prolong the recovery time. Secondly, the sample size of this study is small. We only considered the reduction in laryngospasm (as complication) when calculating the sample size, which may not be adequately powered for other complications. Future studies should consider other complications as well such as coughing, body movements, bronchospasm etc. while calculating the sample size.

## Conclusion

Intranasal dexmedetomidine at 1 μg·kg^− 1^, with its sedative and analgesic effects, reduced the incidences of laryngospasm, breath-holding, and coughing during fiberoptic bronchoscopy for FB removal. Moreover, it reduced postoperative agitation without a prolonged recovery time.

## Data Availability

The datasets used and/or analyzed during the current study are available from the corresponding author on reasonable request.

## Abbreviations

BB: both right and left bronchus
BIS: Bispectral index
DEX group: dexmedetomidine group
EtCO2: end-tidal carbon dioxide
EtSevo: end-tidal sevoflurane
FB: foreign body
HR: heart rate
LB: left bronchus
LMA: laryngeal mask airway
OD: outside diameter
PACU: post-anesthesia care unit
RB: right bronchus
RR: respiratory rate
SpO_2_: oxygen saturation
T: tracheal
